# Prevalence of post-concussion syndrome and associated factors among patients with traumatic brain injury at Debre Tabor Comprehensive Hospital, North Central Ethiopia

**DOI:** 10.3389/fneur.2022.1056298

**Published:** 2022-11-21

**Authors:** Assefa Agegnehu Teshome, Gashaw Walle Ayehu, Getachew Yideg Yitbark, Endeshaw Chekol Abebe, Misganaw Asmamaw Mengstie, Mohammed Abdu Seid, Yalew Melkamu Molla, Nega Dagnaw Baye, Tadeg Jemere Amare, Agmas Wassie Abate, Taklo Semineh Yazie, Kidist Hunegn Setargew

**Affiliations:** ^1^Department of Biomedical Science, College of Health Science, Debre Tabor University, Debre Tabor, Ethiopia; ^2^Department of Pediatrics and Child Health, College of Medicine and Health Science, University of Gondar, Gondar, Ethiopia; ^3^Department of Psychiatry, Dr. Amebachew Memorial Hospital, Tach Gaynt, Ethiopia; ^4^Pharmacology and Toxicology Unit, Department of Pharmacy, College of Health Science, Debre Tabor University, Debre Tabor, Ethiopia; ^5^Addis Zemen Hospital, Addis Zemen, Ethiopia

**Keywords:** post-concussion syndrome, head injury, associated factors, Debre Tabor, traumatic brain injury

## Abstract

**Introduction:**

The occurrence of three or more of the following signs and symptoms, such as headache, dizziness, exhaustion, irritability, sleeplessness, difficulties in concentrating, or memory problems, following a head injury is referred to as post-concussion syndrome (PCS). Even though post-concussion syndrome has not been studied in Ethiopia, the productive age group is frequently affected by health issues related to head trauma, which either directly or indirectly affect the growth of the nation.

**Objective:**

To assess the prevalence and associated factors of post-concussion syndrome among patients with traumatic brain injury at Debre Tabor Comprehensive Hospital, Debre Tabor, North Central Ethiopia.

**Methods:**

A successive sampling technique was used to conduct a hospital-based cross-sectional study on 405 traumatic brain injury patients at Debre Tabor Comprehensive Hospital from January 1, 2022, to May 30, 2022. SPSS version 25 was used to analyze the data. The factors connected to post-concussion syndrome were found using bivariate and multivariable logistic regression analysis. Statistical significance was determined by a *P*-value of ≤ 0.05.

**Results:**

During the data collection period, 405 cases in total were interviewed, with a 98% response rate. More than half (60.7%) of patients were married, with the majority of patients (39.8%) falling between the ages of 18 and 29. At least three post-concussion syndrome symptoms were present in 42.8% of subjects. A history of comorbidities, GCS levels of 8 or below, 9 to 12 at the time of presentation, brain neuroimaging findings, and having fair or poor social support were found to be substantially linked with PCS in multivariate logistic regression.

**Conclusion:**

About 41.5% of study participants had at least three symptoms of PCS. The Glasgow coma scale level at the time of presentation, the reason for the injury, social support, and the site of the injury were all significantly associated with the occurrence of PCS.

## Introduction

The World Health Organization (WHO) defines post-concussion syndrome (PCS) as the occurrence of three or more of the signs and symptoms listed below following a head injury such as headache, dizziness, exhaustion, irritability, sleeplessness, difficulties concentrating, or memory problems (1). The symptoms of post-concussion syndrome (PCS) may persist for weeks, months, or even a year or more following a concussion (2, 3). Approximately 34–35% of concussion victims have ongoing or long-standing symptoms 3–6 months after the incident (4). Having concussion symptoms for more than four weeks after the initial injury in children, and for weeks or months in adults is referred to as a prolonged concussion (5).

A conclusive test does not exist for post-concussion syndrome. The primary factors in the diagnosis include a history of head trauma and reported symptoms. To assess symptoms, a physical examination and potentially a head CT or MRI scan may be performed. To rule out further causes of symptoms, such as infection, bleeding into the brain, or poisoning, additional tests may be performed (6–8). Despite the lack of a specific treatment for PCS, medications, physical, and behavioral therapy can help resolve symptoms. It is crucial to provide information regarding symptoms and recovery expectations (9).

According to estimates, 40–80% of the 2 million Americans who experience a minor head injury (MHI) each year go on to develop post-concussive syndrome (PCS), which can cause them to miss work or school and impair their social behavior (10).

One of the most frequent consequences of TBI is post-concussion syndrome (PCS), which consists of somatic, cognitive, and emotional symptoms (6). Studies on PCS have revealed significant variations in the syndrome's prevalence, ranging from <5% (11) to 58% (12), while 10–15% of concussions are most frequently observed. The disagreement over the definition of PCS is a key factor in this wide range. However, there is a significant chance that PCS will be misdiagnosed because the majority of its symptoms are either common or could be made worse by other illnesses. For instance, headaches following a concussion could resemble migraines or tension headaches (13).

Age, gender, expectations of disability, and somatic illnesses or mental illnesses are a few risk factors that have been linked to PCS. The development of PCS is thought to be influenced by physiological and psychological occurrences before, during, and after the injury. A long-running controversy surrounds PCS's cause, which is still being discussed today. Some experts claim that post-concussion symptoms are brought on by structural damage to the brain or disruption of neurotransmitter systems. However, radiological evidence of brain damage was absent in 38% of people who experienced concussion-like symptoms following a head injury (14, 15).

The prevalence of a group of physical, mental, emotional, and behavioral symptoms that can be identified in victims of traumatic brain injury ranges from 11 to 64% (16). It is not known how common persistent symptoms are after a year, although it is assumed to be 5%. Although PCS is prevalent, there are issues regarding its accuracy since children typically exhibit behavioral abnormalities after any accident and because circumstances that existed prior to the damage, as well as medical and legal concerns after the injury, may have an impact on recovery (17). Up to 60% of individuals with mild traumatic brain injury (MTBI) may experience the post-concussion syndrome (PCS), which is characterized by the persistence of symptoms such as headaches, fatigue, memory loss, and emotional instability (18).

Numerous international studies have shown that brain injuries are a common health problem that mostly affects the world's productive age groups and greatly increases medical costs. Most commonly impacting the population's productive age group and contributing significantly to morbidity and death in Africa, head injuries have negative economic effects on both individuals and the continent's community (19).

Although the limited study on post-concussion syndrome in Ethiopia, it is a common health problem that affects the productive age group and either directly or indirectly affects the development of the country (15). Because the issue affects the country's most productive age group, it is crucial to focus on doing this study on the prevalence and associated factors of post-concussion syndrome.

## Methods

### Study design, study area and study period

A hospital-based based cross-sectional study was conducted in Emergency department of Debre Tabor Comprehensive (DTCH), from January 1, 2022 to May 30, 2022, which is found in Debre Tabor city and found 665 km from Addis Ababa (the capital city of Ethiopia). The hospital is the largest in the South Gondar zone, which was established in 1953 and serves more than 2.5 million population in its catchment area. With 160 inpatient beds across five major departments, the hospital has more than 40 specialists in a variety of medical specialties along with 250 additional health workers.

### Source and study population

The source population consisted of all patients who presented to the adult emergency department of DTCH with head injury and the study population was made up of all patients who had traumatic brain injuries and had visited the adult emergency department of DTCH throughout the study period.

### Inclusion and exclusion criteria

Patients who were seriously ill or unconscious during the data collection period were excluded from the study, while patients with traumatic brain injuries who were 18 years of age or older were all included.

### Sample size determination and sampling techniques

The required sample size was determined using the single population proportion formula by taking the prevalence of PCS (41.5%) among head injury patients from a study done at the emergency department of Hawassa University Comprehensive and Specialized Hospital (15), with a 95% level of confidence and 5% margin of error.


$$\begin{array}{l} \text{N=}(\text{Z}\alpha /2{)}^{2}\text{p}(\text{p1-p}){\text{/d}}^{2}\text{n=}(1.96{)}^{2}0.415(1-0.415)/0.05^{\text{2}}\\ \;=368 \end{array}$$

Adding a 10% non-response rate. The final sample size was 405. A consecutive sampling technique was used to select the respondents for their participation.

### Data collection tool and procedures

Data on the prevalence of PCS were gathered using a standard questionnaire. The most widely used tool is the Rivermead Post-Concussion Symptoms Questionnaire (RPQ), which has been shown to have validity and reliability (20, 21). With consideration for the high baseline incidence of symptoms, this questionnaire asks patients to rank the severity of each of 16 symptoms in comparison to before the mild traumatic brain injury (MTBI). Face-to-face interviews at appointed follow-ups during the first 12 months after injury were undertaken. The interview covered details on sociodemographic factors, clinical traits, and behavioral facets. Two medical intern students who were working in the emergency room were participated in collected the data. For ensuring the validity of the data, the interviewees were questioned in Amharic, their native tongue.

### Data quality control measures

Trained medical interns gathered the data. The questionnaire was pre-tested on 5% of the study participants before the actual data-collecting period to make sure it was clear, comprehensible, and complete. The results of the final analysis did not include the pre-test data.

### Data processing and analysis

Before being subjected to SPSS version 25 analysis, the data was double-checked for accuracy, coded, and entered into Epi-info version 7.2. Using descriptive statistical analysis, the estimated frequencies and percentages of the variables were computed. To investigate the link between the outcome and the explanatory variables, analyses of multivariate and bivariate logistic regression were performed. A *P*-value of 0.05 or less was regarded as statistically significant, and an odds ratio with a 95% confidence interval was used to assess the strength of associations.

## Results

### Socio-demographic characteristics

Out of 405 selected traumatic brain injury patients, 397 patients have participated in this study with a response rate of 98%. Two hundred eighty-three (71.3%) were male patients. The patients' mean (standard deviation) age was 34.4 (16.7) years. About 241 (60.7%) of the patients were married, and only 47 (11.8%) were unable to read and write. Most of the patients (318, 80.1%) were orthodox religious followers, and more than two-thirds (298, 75.1%) were from rural residences (Table 1).

**Table 1:** Socio-demographic characteristics of study participants attending emergency outpatient department of DTCH, Debre Tabor, Ethiopia (*n* = 405).

**Characteristics**	**Categories**	**Number**	**Percent**
Age	18–29	158	39.8
	30–39	145	36.5
	40–49	61	15.4
	50+	33	8.3
Sex	Male	283	71.3
	Female	114	28.7
Religion	Orthodox	318	80.1
	Muslim	59	14.9
	Protestant	14	3.5
	Other	6	1.5
Marital status	Single	127	32
	Married	241	60.7
	Divorced	11	2.8
	Widowed	18	4.5
Occupation	Government employee	104	26.2
	Merchant	92	23.2
	Farmer	57	14.3
	House wife	44	11.1
	Student	64	16.1
	Daily labor	36	9.1
Educational status	Unable to read and write	47	11.8
	Able to read and write	41	10.3
	Primary	71	17.9
	Secondary	110	27.7
	College and above	128	32.2
Place of residence	Rural	298	75.1
	Urban	99	24.9

### Clinical characteristics

Concerning the duration of illness, 67.5% of study participants were sick for 1 week, and 31.0% were injured in the right lateral part of their brain. Regarding social support, 28.0% of respondents have very good social support. In terms of injury causes, 39.3% of study participants were injured in a car accident (Table 2).

**Table 2:** Characteristics of injures of study participants attending emergency outpatient department of DTCH, Debre Tabor, Ethiopia (*n* = 405).

**Characteristics**	**Categories**	**Number**	**Percent**
Duration of illness	< 1 week	268	67.5
	1–2 weeks	76	19.2
	3–4 weeks	37	9.3
	>1 month	16	4
Localization of injury	Right lateral	123	31
	Left lateral	118	29.7
	Frontal	115	29
	Occipital	41	10.3
Clinical presentation at time of admission	Shock	74	18.6
	Unconsciousness	148	37.3
	Bleeding	127	32
	0ther	48	12.1
Length of stay in hospital	< 5 days	167	42.1
	1 week	112	28.2
	2–4 weeks	87	21.9
	5+ weeks	31	7.8
Duration of treatment	< 1 week	161	40.5
	1–5 weeks	148	37.3
	6–10 weeks	67	16.9
	10+ weeks	21	5.3
GCS at time of presentation	≤ 8	39	9.8
	12-Sep	119	30
	13+	239	60.2
Brain neuroimaging findings			
	Diffuse axonal injury	42	10.6
	Sub dural hematoma	129	32.5
	Epi dural hematoma	148	37.3
	Others	78	19.6
The types of intervention given	Non-operative approach	295	74.3
	Operative approach (surgery)	102	25.7
History of comorbidities	Yes	69	17.4
	No	328	82.6

### Prevalence and factors associated with PCS

**Figure 1:**
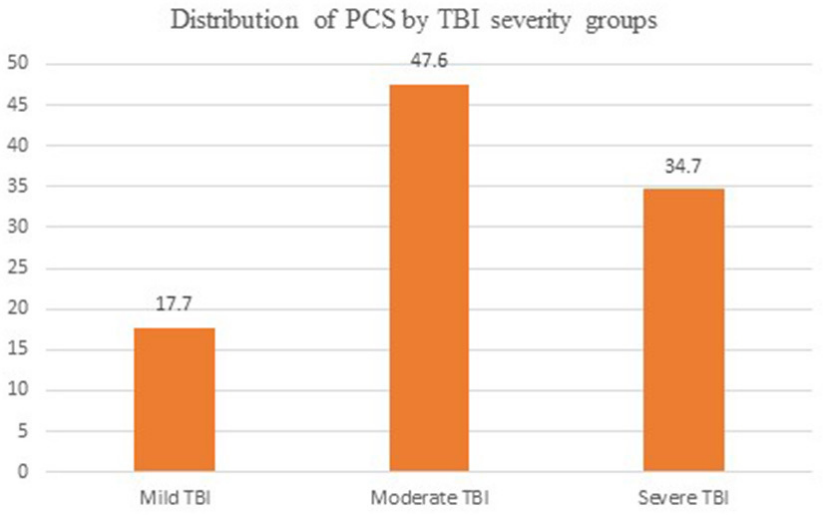
Distributions of PCS by TBI severity groups by percentage. PCS, post-concussion syndrome; TBI, traumatic brain injury.

In this study, the overall prevalence of post-concussion syndrome was 42.8%. Moderate traumatic brain injury accounts 47.6% of post-concussion syndrome (Figure 1). In the bivariate analysis, having a history of comorbidities, GCS values of eight or less, 9–12 at time of presentation, trauma features and having fair and poor social support were identified to be significantly associated with PCS. Likewise, during multivariate logistic regression, the above variables are significantly associated with PCS. Head injury patients with fair or poor [AOR = 2.51; 95% CI (1.37–3.83)] social support were 2.5 times more likely to have PCS than those with strong social support. Head injury patients who have a history of comorbidities [AOR = 1.79; 95% CI (1.04–2.76)] were 1.8 times more likely to have PCS than those who do not. At the time of presentation, patients with a GCS of 8 or less (AOR = 3.75; 95% CI (2.09–8.41) or 9-12 (AOR = 3.42; 95% CI (2.24–5.77) were 3.75 and 3.42 times more likely to develop PCS than patients with a GCS of 13+. Traumatic brain injury patients who had brain neuroimaging findings of Diffuse axonal injury, subdural hematoma and epidural hematoma [AOR = 15.34; 95% CI (5.76–39.73)], [AOR = 5.25; 95% CI (2.16–10.64)], [AOR = 8.53; 95% CI (3.92–21.32)] were 15.34, 5.25 and 8.53 times more likely to have PCS than those who have other findings, respectively (Table 3).

**Table 3:** Factors associated PCS (bivariate and multivariate logistic regression) of study participants attending emergency outpatient department of Debre Tabor Compressive Hospital, Debre Tabor, Ethiopia (*n* = 397).

**Variable**	**PC**		**COR (95% CI)**	**AOR (95% CI)**
	**Yes**	**No**		
**Age**				
18–29	69	89	1	1
30–39	61	84	0.94 (0.59–1.48)	0.87 (0.54–1.38)
40–49	26	35	0.96 (0.53–1.74)	0.91 (0.48–1.57)
50+	14	19	0.95 (0.44–2.03)	0.87 (0.38–1.97)
**Sex**				
Male	129	154	1.49 (0.95–2.33)	1.53 (0.89–2.17)
Female	41	73	1	1
**Marital status**				
Single	55	72	1	1
Married	107	134	1.04 (0.68–1.61)	1.03 (0.65–1.45)
Divorced/Widowed	8	21	0.50 (0.21–1.21)	0.47 (0.19–1.16)
**Occupation**				
Government employee	42	62	1	1
Merchant	37	55	0.99 (0.56–1.76)	0.92 (0.53–1.71)
Farmer	28	29	1.42 (0.74–2.73)	1.39 (0.71–2.56)
House wife	17	27	0.93 (0.45–1.91)	0.89 (0.41–1.87)
Student	29	35	1.22 (0.65–2.29)	1.19 (0.57–2.19)
Daily labor	17	19	1.32 (0.62–2.83)	1.28 (0.57–2.74)
**Education**				
Unable to read and write	26	21	1	1
Able to read and write	17	24	0.57 (0.24–1.33)	0.53 (0.14–1.25)
Primary	32	39	0.86 (0.40–1.88)	0.83 (0.36–1.81)
Secondary	41	69	0.72 (0.39–1.33)	0.67 (0.31–1.28)
College and above	54	74	0.86 (0.48–1.55)	0.82 (0.43–1.51)
**Social support**				
Strong	37	78	1	1
Moderate	43	76	1.19 (0.69–2.05)	1.16 (0.63–2.01)
Fair/Poor	90	73	2.69 (1.58–4.28)^**^	2.51 (1.37–3.83)^**^
**Cause of head injury**				
Road traffic accident	74	82	1	1
Interpersonal fights	52	70	0.82 (0.51–1.33)	0.78 (0.46–1.29)
Falling down	23	47	0.54 (0.03–0.98)^*^	0.49 (0.02–0.96)^*^
Other	21	28	0.83 (0.43–1.59)	0.78 (0.41–1.55)
**Localization of injury**				
Right lateral	54	69	1	1
Left lateral	52	66	1.01 (0.60–1.67)	0.98 (0.51–1.69)
Frontal	55	60	1.02 (0.61–1.72)	1.01 (0.57–1.68)
Occipital	9	32	0.36 (0.16–0.82)^*^	0.32 (0.12–0.76)^*^
**History of comorbidities**				
Yes	38	31	1.82 (1.08–3.07)^**^	1.79 (1.04–2.76)^**^
No	132	196	1	1
**GCS at time of presentation**				
≤ 8	25	14	4.31 (2.12–8.78)^**^	3.75 (2.09–8.41)^**^
9–12	75	44	4.11 (2.58–6.55)^**^	3.42 (2.24–5.77)^*^
13+	70	169	1	1
**Brain neuroimaging findings**				
Diffuse axonal injury	29	13	19.52 (7.31–52.08) ^**^	15.34 (5.76–39.73) ^**^
Sub Dural hematoma	51	78	5.72 (2.54–12.89) ^**^	5.25 (2.16–10.64) ^**^
Epi Dural hematoma	82	66	10.87 (4.88–24.20) ^**^	8.53 (3.92–21.32) ^**^
Others	8	70	1	1

AOR adjusted odds ratio, CI confidence interval, COR crude odds ratio,

* *P* < 0.05,

** *P* < 0.02, other (skull fracture related problems).

## Discussion

The dorsal raphe nucleus is one of the crucial circuitry systems in the brain that is probably harmed by concussion. Within the brainstem, the serotenergic fiber tracts are under the direction of the dorsal raphe nucleus. Due to their far-reaching projections, the fiber tracts are vulnerable to damage and shearing during the impact of a concussion (22, 23).

In this study, post-concussion syndrome was prevalent in 42.8, or 95% CI (36.7–45.4%) among head injury patients who visited the DTCH emergency room. While the results of this study were more significant, a study conducted a CENTER-TBI (the Collaborative European Neurotrauma Effectiveness Research) found that PCS symptoms were present in 58% of the subjects (24). Additionally, it was much greater than studies done in Malaysia, which discovered 34% of patients who survived mild TBI acquired PCS (25). The fact that the present study's study location, sample size, and socioeconomic makeup differ from the one previously stated could be the cause of the discrepancy. On the other hand, this outcome was equivalent to the 41.5% (15) at the comprehensive specialized hospital of Hawassa University in Ethiopia.

According to this study, the majority of injuries were caused by interpersonal conflicts (122, 30.7%) and road traffic accidents (156, 39.3%). Interpersonal conflicts accounted for 38.5% of head injury cases at Jimma University Hospital in Southwest Ethiopia, while road traffic accidents accounted for 36.5% of those cases (26) and a study from Gondar University Referral Hospital in Northwest Ethiopia also revealed findings that were similar to these (27). Contradicting this report, a study conducted in Australia found that the majority of injuries resulted from sporting and recreational activities (28). This variation may be due to the difference in socio-cultural and behavioral characteristics of the study participants.

In comparison to patients who sustained right lateral injuries, those with occipital brain injuries had a 32% decreased likelihood of developing PCS. A study conducted at the Hawassa University Comprehensive Specialized Hospital in southern Ethiopia also lends credence to this result (15). additionally, a follow-up study conducted in the United States discovered that participants with frontotemporal and parietal head injuries had more severe effects and atrophied more rapidly than those with lesions to other regions of the brain (29).

The current study found that social support was a significant factor in the development of post-concussion syndrome, which is consistent with the scientific justification of the neuropsychiatrist's theory that patients with fair or poor social support were more likely to develop PCS than patients with strong social support. Those with fair or poor social support had 2.47 and 2.62 times the likelihood of developing PCS compared to patients with excellent social support after a head injury. Other research carried out at the Hawassa University Comprehensive Specialized Hospital in southern Ethiopia does not agree with this report. As was shown, 41.1% (15) of study participants who experienced PCS had just fair to poor social support.

Patients with head injuries and GCS values of eight or less and 9–12 at the time of presentation were 3.75 (AOR = 3.75, 95% CI, 2.09–8.41) and 3.42 (AOR = 3.42, 2.24–5.77) times more likely to develop PCS, respectively, than patients with head injuries and GCS values of 13 or higher. This conclusion is reinforced by the fact that the GCS is still an important tool for neurological evaluation following a head injury and that, in the majority of studies, the classification of the trauma's severity is still dependent on the admission GCS (15, 30). A score of eight or less is required to distinguish between a serious head injury and a moderate to mild head injury; in this study, the development of PCS increases with the severity of the head injury, in line with findings in a one-year prospective study done from April 21, 2006-April 20, 2007 in Nigeria's new neurosurgical centers (31).

## Conclusion

About 42.8% of those who took part in the study showed at least three PCS symptoms. The Glasgow coma scale level at the time of presentation, the reason for the injury, social support, and the site of the injury were all significantly associated with the occurrence of PCS.

### Recommendation

Future research ought to take into account the imaging findings of people who have suffered head injuries as supporting evidence in addition to evaluating the symptoms listed in the PCS tool.

### Limitation

The cross-sectional nature of the study precludes the inference of causality.

## Data availability statement

The raw data supporting the conclusions of this article will be made available by the authors, without undue reservation.

## Ethics statement

The studies involving human participants were reviewed and approved by the Ethics Review Committee of the College of Health Science at Debre Tabor University. The patients/participants provided their written informed consent to participate in this study.

## Author contributions

MM, MS, AA, TA, and EA participated in the conception, design of the study, reviewing the proposal, data analysis, and report writing. NB, GA, YM, GY, TY, and KS were participated in data analysis and report writing. AT performed data analysis and prepared the manuscript for publication. All authors read and approved the final manuscript.

## Conflict of interest

The authors declare that the research was conducted in the absence of any commercial or financial relationships that could be construed as a potential conflict of interest.

## Publisher's note

All claims expressed in this article are solely those of the authors and do not necessarily represent those of their affiliated organizations, or those of the publisher, the editors and the reviewers. Any product that may be evaluated in this article, or claim that may be made by its manufacturer, is not guaranteed or endorsed by the publisher.

## Abbreviations

AOR: Adjusted odds ratio
CI: Confidence Interval
DTCH: Debre Tabor Comprehensive Hospital
GCS: Glasgow Coma Scale
MTBI: minor traumatic brain injury
MHI: minor head injury
PCS: Post -concussion syndrome
RPQ: Rivermead Post-Concussion Symptoms Questionnaire
SD: Standard deviation
SPSS: Stastical Package for Social Science
WHO: World Health Organization.
